# Supplementary material for: Is Piperacillin-Tazobactam an Appropriate Empirical Agent for Hospital-Acquired Sepsis and Community-Acquired Septic Shock of Unknown Origin in Australia?
Source: Healthcare (Basel). 2022 May 5;10(5):851. doi: 10.3390/healthcare10050851 (PMC9142067; doi:10.3390/healthcare10050851)
Supplement: Supplementary file 1 [file healthcare-10-00851-s001.zip › healthcare-1684234-supplementary.pdf]

Table S1. Additional case reports addressing the clinical efficacy of TZP as monotherapy in sepsis

Reference	Time period	Setting	Study design	Sample size	Causative pathogen	Key findings	Limitations
**Gram-negative aerobes**							
Yokota 2012[1]	2012	Japan	Case report	1	*Raoultella planticola*	A patient with acute cholangitis achieved clinical improvement with high dose definitive TZP therapy.	Case report; No data on mortality, as patient subsequently transferred hospitals
Ahsan 2019[2]	2019	USA	Case report	1	*Chryseobacterium* spp.	A septic haemodialysis patient relapsed on ampicillin-sulbactam and responded well to TZP.	Case report
Yaita 2013[3]	2011	Japan	Case report	1	*P. aeruginosa*	Patient with liver abscesses developed a new liver abscess from which TZP-resistant *P. aeruginosa* was isolated.	Case report
Sohn 2015[4]	2015	Korea	Case report	1	*Delftia lacustris*	Septic patient with phaeochromocytoma. Clinical and microbiological resolution with two weeks of TZP.	Case report
**Anaerobes**							
Hung 2014[5]	2014	Taiwan	Case report	1	*Clostridium innocuum*	An immunocompromised patient recovered with two weeks of TZP.	Case report
Hans 2016[6]	2016	USA	Case report	1	*Pediococcus pentosaceus*	Sepsis secondary to complicated urinary tract infection resolved with ten days of TZP.	Case report

Table S2. Additional case report relating to clinical outcomes related to TZP pharmacokinetic and dosing considerations in sepsis

Reference	Time period	Setting	Study design	Sample size	Key findings	Limitations
D'Agostino 2015[7]	2015	USA	Case report	1	In a patient with *Elizabethkingia meningosepticum* bacteraemia, switch from standard to high dose extended infusion resulted in microbiological clearance. No clinical improvement, however, and patient transferred to hospice.	Case report

References

1. Yokota K, Gomi H, Miura Y, Sugano K, Morisawa Y. Cholangitis with septic shock caused by Raoultella planticola. Journal of medical microbiology. 2012;61(Pt 3):446-9.
2. Ahsan MJ, Ahmad S, Latif A, Reddy JT. Chryseobacterium spp-associated bacteraemia in a haemodialysis patient: a diagnostic challenge. BMJ case reports. 2019;12(11).
3. Yaita K, Sameshima I, Takeyama H, Matsuyama S, Nagahara C, Hashiguchi R, et al. Liver abscess caused by multidrug-resistant Pseudomonas aeruginosa treated with colistin; a case report and review of the literature. Internal medicine (Tokyo, Japan). 2013;52(12):1407-12.
4. Sohn KM, Baek J-Y. Delftia lacustris septicemia in a pheochromocytoma patient: case report and literature review. Infectious diseases (London, England). 2015;47(5):349-53.
5. Hung Y-P, Lin H-J, Wu C-J, Chen P-L, Lee J-C, Liu H-C, et al. Vancomycin-resistant Clostridium innocuum bacteremia following oral vancomycin for Clostridium difficile infection. Anaerobe. 2014;30(9505216):24-6.
6. Han A, Mehta J, Pauly RR. Septic Shock Secondary to a Urinary Tract Infection with Pediococcus Pentosaceus. Missouri medicine. 2016;113(3):179-81.
7. D'Agostino C, Rhodes NJ, Skoglund E, Roberts JA, Scheetz MH. Microbiologic clearance following transition from standard infusion piperacillin-tazobactam to extended-infusion for persistent Gram-negative bacteremia and possible endocarditis: A case report and review of the literature. Journal of infection and chemotherapy : official journal of the Japan Society of Chemotherapy. 2015;21(10):742-6.
